# Supplementary material for: The impact of genetic risk for Alzheimer’s disease on the structural brain networks of young adults
Source: Front Neurosci. 2022 Nov 30;16:987677. doi: 10.3389/fnins.2022.987677 (PMC9748570; doi:10.3389/fnins.2022.987677)
Supplement: Supplementary file 1 [file Data_Sheet_1.docx]

**Supplementary Material**

**A. Permutation Tests**

Below we give the *p*-values that resulted from the permutation testing, as described in Section 2.7. We report on the cases in which the correlations between graph theoretical metrics and PRS were statistically significant.

Permutation-corrected *p*-values for the correlation between mean nodal strength of the NS-weighted DMN and genome-wide PRS incl. APOE:

P_T_=0.3: 0.0438

Permutation-corrected *p*-values for the correlation between mean nodal strength of the NS-weighted DMN and genome-wide PRS excl. APOE:

P_T_=0.3: 0.0471

Permutation-corrected *p*-values for the correlation between mean betweenness centrality of the FA-weighted DMN and activation of the immune response PRS incl. APOE:

P_T_=0.01: 0.0083

Permutation-corrected *p*-values for the correlation between mean betweenness centrality of the FA-weighted DMN and activation of the immune response PRS excl. APOE:

P_T_=0.01: 0.0209

Permutation-corrected *p*-values for the correlation between mean nodal strength of the NS-weighted visual network and genome-wide PRS incl. APOE:

P_T_=0.1: 0.0062; P_T_=0.3: 0.0036; P_T_=0.5: 0.0014

Permutation-corrected *p*-values for the correlation between mean nodal strength of the NS-weighted visual network and genome-wide PRS excl. APOE:

P_T_=0.1: 0.0271; P_T_=0.3: 0.0093; P_T_=0.5: 0.0031

Permutation-corrected *p*-values for the correlation between mean clustering coefficient of the NS-weighted visual network and tau protein binding PRS incl. APOE:

P_T_=0.3: 0.0155; P_T_=0.5: 0.0157

Permutation-corrected *p*-values for the correlation between the mean betweenness centrality of the NS-weighted visual network and the plasma lipoprotein particle assembly PRS incl. APOE:

P_T_=0.3: 0.0102; P_T_=0.5: 0.0055

Permutation-corrected *p*-values for the correlation between the mean betweenness centrality of the NS-weighted visual network and the plasma lipoprotein particle assembly PRS excl. APOE:

P_T_=0.3: 0.0772; P_T_=0.5: 0.0333

Permutation-corrected *p*-values for the correlation between the rich-club connectivity of the NS-weighted whole-brain network and the genome-wide PRS incl. APOE:

P_T_=0.3: 0.0184; P_T_=0.5: 0.0346

Permutation-corrected *p*-values for the correlation between the rich-club connectivity of the NS-weighted whole-brain network and the genome-wide PRS excl. APOE:

P_T_=0.3: 0.0250; P_T_=0.5: 0.0492

Permutation-corrected *p*-values for the correlation between the feeder connectivity of the NS-weighted whole-brain network and the genome-wide PRS incl. APOE:

P_T_=0.3: 0.0291; P_T_=0.5: 0.0365

Permutation-corrected *p*-values for the correlation between the feeder connectivity of the NS-weighted whole-brain network and the genome-wide PRS excl. APOE:

P_T_=0.3: 0.0637; P_T_=0.5: 0.0463

**B. Correlation values for the different thresholds on the number of streamlines**

The tables below give the correlation coefficients and the *p*-values for the different values of NS_thr_, where NS_thr_ is the maximum value of NS for tracts excluded from the brain network. For the case of the correlations not listed, the differences between the correlation coefficients / *p*-values for the 12 NS_thr_ were on the third significant digit, and for that reason we do not list them here.

Correlation between the mean betweenness centrality of the DMN and the activation of the immune response PRS (incl. APOE) for the FA-weighted networks, for P_T_=0.01:

| **NS_thr_** | **1** | **2** | **3** | **4** | **5** | **6** | **7** | **8** | **9** | **10** | **11** | **12** |
| --- | --- | --- | --- | --- | --- | --- | --- | --- | --- | --- | --- | --- |
| **Correlation coefficient** | -0.16 | -0.17 | -0.17 | -0.18 | -0.16 | -0.16 | -0.17 | -0.18 | -0.17 | -0.16 | -0.17 | -0.16 |
| ***p*-value** | 3x10^-4^ | 10^-4^ | 9x10^-5^ | 5x10^-5^ | 1x10^-4^ | 2x10^-4^ | 2x10^-4^ | 7x10^-5^ | 2x10^-4^ | 3x10^-4^ | 2x10^-4^ | 3x10^-4^ |

Correlation between the mean clustering coefficient of the visual network and the tau protein binding PRS (incl. APOE) for the NS-weighted networks, for P_T_=0.3 and 0.5:

| **NS_thr_** | | **1** | **2** | **3** | **4** | **5** | **6** | **7** | **8** | **9** | **10** | **11** | **12** |
| --- | --- | --- | --- | --- | --- | --- | --- | --- | --- | --- | --- | --- | --- |
| **P_T_=0.3** | **Cor Coef** | -0.12 | -0.12 | -0.11 | -0.14 | -0.14 | -0.13 | -0.12 | -0.13 | -0.13 | -0.14 | -0.12 | -0.13 |
|  | ***p*-value** | 7x10^-3^ | 6x10^-3^ | 10^-2^ | 2x10^-3^ | 10^-3^ | 2x10^-3^ | 6x10^-3^ | 3x10^-3^ | 2x10^-3^ | 2x10^-3^ | 9x10^-3^ | 4x10^-3^ |
| **P_T_=0.5** | **Cor Coef** | -0.12 | -0.11 | -0.12 | -0.14 | -0.14 | -0.13 | -0.12 | -0.13 | -0.13 | -0.12 | -0.12 | -0.13 |
|  | ***p*-value** | 7x10^-3^ | 10^-2^ | 8x10^-3^ | 2x10^-3^ | 10^-3^ | 2x10^-3^ | 7x10^-3^ | 3x10^-3^ | 2x10^-3^ | 6x10^-3^ | 7x10^-3^ | 4x10^-3^ |

Correlation between the rich club connectivity of the whole-brain network and the genome-wide PRS (incl. APOE) for the NS-weighted networks, for P_T_=0.3 and 0.5:

| **NS_thr_** | | **1** | **2** | **3** | **4** | **5** | **6** | **7** | **8** | **9** | **10** | **11** | **12** |
| --- | --- | --- | --- | --- | --- | --- | --- | --- | --- | --- | --- | --- | --- |
| **P_T_=0.3** | **Cor Coef** | -0.16 | -0.16 | -0.16 | -0.16 | -0.16 | -0.16 | -0.16 | -0.16 | -0.16 | -0.16 | -0.15 | -0.15 |
|  | ***p*-value** | 4x10^-4^ | 4x10^-4^ | 4x10^-4^ | 4x10^-4^ | 4x10^-4^ | 4x10^-4^ | 4x10^-4^ | 4x10^-4^ | 4x10^-4^ | 4x10^-4^ | 10^-3^ | 10^-3^ |
| **P_T_=0.5** | **Cor Coef** | -0.14 | -0.14 | -0.14 | -0.14 | -0.14 | -0.14 | -0.14 | -0.14 | -0.14 | -0.14 | -0.15 | -0.15 |
|  | ***p*-value** | 10^-3^ | 10^-3^ | 10^-3^ | 10^-3^ | 10^-3^ | 10^-3^ | 10^-3^ | 10^-3^ | 10^-3^ | 10^-3^ | 10^-3^ | 10^-3^ |

Correlation between the feeder connectivity of the whole-brain network and the genome-wide PRS (incl. APOE) for the NS-weighted networks, for P_T_=0.3 and 0.5:

| **NS_thr_** | | **1** | **2** | **3** | **4** | **5** | **6** | **7** | **8** | **9** | **10** | **11** | **12** |
| --- | --- | --- | --- | --- | --- | --- | --- | --- | --- | --- | --- | --- | --- |
| **P_T_=0.3** | **Cor Coef** | -0.14 | -0.14 | -0.14 | -0.14 | -0.14 | -0.14 | -0.14 | -0.14 | -0.14 | -0.14 | -0.12 | -0.12 |
|  | ***p*-value** | 10^-3^ | 10^-3^ | 10^-3^ | 10^-3^ | 10^-3^ | 10^-3^ | 10^-3^ | 10^-3^ | 10^-3^ | 10^-3^ | 5x10^-3^ | 5x10^-3^ |
| **P_T_=0.5** | **Cor Coef** | -0.15 | -0.15 | -0.15 | -0.15 | -0.15 | -0.15 | -0.15 | -0.15 | -0.15 | -0.15 | -0.13 | -0.13 |
|  | ***p*-value** | 9x10^-4^ | 9x10^-4^ | 9x10^-4^ | 9x10^-4^ | 9x10^-4^ | 9x10^-4^ | 9x10^-4^ | 9x10^-4^ | 9x10^-4^ | 9x10^-4^ | 2x10^-3^ | 2x10^-3^ |
